# Non-enzymatic glucosylation induced neo-epitopes on human serum albumin: A concentration based study

**DOI:** 10.1371/journal.pone.0172074

**Published:** 2017-02-13

**Authors:** Km Neelofar, Zarina Arif, Jamal Ahmad, Khursheed Alam

**Affiliations:** 1 Rajiv Gandhi Centre for Diabetes and Endocrinology, J.N. Medical College, Aligarh Muslim University, Aligarh, Uttar Pradesh, India; 2 Department of Biochemistry, J.N. Medical College, Aligarh Muslim University, Aligarh, Uttar Pradesh, India; Islamic Azad University Mashhad Branch, ISLAMIC REPUBLIC OF IRAN

## Abstract

Hyperglycaemia induced non enzymatic glycation is accelerated in diabetic patients and aggressively involved in diabetes progression. Human serum albumin (HSA) is the most abundant protein in blood circulation. In hyperglycaemia, it undergoes fast glycation and results in the impairment of structure. Our previous study has demonstrated structural alterations in Amadori-albumin modified with different glucose concentrations from physiological to pathophysiological range. Here, we focused on immunological characterization of Amadori-albumin. Immunogenicity of Amadori-albumin was analysed by direct binding and competitive ELISA. Amadori-albumin was found to be highly immunogenic (expect albumin modified with 5mM) and induced high titre antibodies depending upon the extent of modification. Very high titre antibodies were obtained with albumin modified with 75mM glucose as compared to native albumin. Anti-Amadori-albumin-IgG from rabbit sera exhibited increased recognition of Amadori-albumin than native albumin in competitive immunoassay. Alteration induced in albumin after glucosylation has made it highly immunogenic. Induced antibodies were quite specific for respective immunogens but showed cross-reaction with other Amadori/native proteins. It suggests that glucosylation has generated highly immunogenic epitopes on albumin. Formation of high molecular weight immune complex with retarded mobility further supports specificity of anti-Amadori-albumin-IgG towards Amadori-albumin. It may be concluded that due to early glycation, an array of modification occurred in HSA structure. Such gross structural changes might favour polymerization of most of the native epitopes into potent immunogenic neo-epitopes, but some original epitopes were still active and has contributed in the immunogenicity. It could be concluded that induction of anti-Amadori-albumin antibodies may be due to protection of glucose modified albumin from protiolytic breakdown. We assumed that this type of protein modifications might occur in diabetic patients in hyperglycaemic conditions that may be recognised as foreign molecules and can induce autoantibodies. Increased level of anti-Amadori-albumin autoantibodies may be used as a biomarker in disease diagnosis and its progression.

## Introduction

Human serum albumin (HSA) is most abundant serum protein. Structurally, it is single chain globular protein with 585 amino acids, contains 1 free cysteine, 1 tryptophan, 59 lysine and other amino acid residues [1]. The crystal structure of HSA shows that it is a three domain, heart shaped molecule. It is a multifunctional protein in human blood and plays an important protective role as an antioxidant [2]. The epsilon amino group lysine and arginine and free amino group of proteins can be non-enzymatically attached to the reducing sugar to form Schiff base which via intermolecular rearrangement forms stable, covalently bonded Amadori products and finally converted into advanced glycation end products (AGEs). This process occurs in individuals with normal plasma glucose concentrations, but HSA is typically 2–3 times more glycated than the rest of the serum proteins in hyperglycaemic condition [3]. Sustained hyperglycaemia leads to glycation of serum proteins preferably at epsilon amino group of lysine residues. Serum glycated proteins represent a potential marker for hyperglycaemia in diabetes mellitus and its complications [4]. Proteins modification in diabetes may lead to Amadori as well as AGEs. The glycation of HSA may have a variety of important physiological effects and the *in vitro* modifications of protein by glucose is regarded as an appropriate model for changes in structure and function relevant to diabetes mellitus [5–7]. Proteins interactions with ligands changed their secondary and tertiary structure that was determined by various techniques [8–9]. Structural stability is the main factor to carry out all its functions otherwise it can involve in diseases progression [10–11]. Such modifications on proteins may lead to generation of neo-epitopes which could in turn be more immunogenic [12]. Immunogenic properties of proteins have been widely used to study their structure. Many research articles have revealed that proteins upon glycation have become immunogenic because of conformational changes that gave the titre of antibodies against the modified protein when injected in experimental animals [13–14]. Glycated poly-L-lysine has been used as an antigen to induce antibodies in experimental animal and was reported to be highly immunogenic and specific towards the corresponding antigen [15].

HSA was therefore incubated in vitro at protein concentration (1 mg/ml) and with a range of glucose concentrations found physiologically in normal (5mM), diabetic plasma (25mM & 50mM) and 75mM non-physiological. Our previous finding has shown that early glycation induced significant structural changes in HSA which is corresponding to glucose concentrations upon early glycation [16]. Now, we hypothesize that due to structural impairment, neo-epitopes may be generated on Amadori-albumin rendering it highly immunogenic compared to native albumin and induced highly specific antibodies in experimental rabbits. The aim of this study was to evaluate the effect of different concentrations of glucose on immunogenic potential of HSA upon early glycation. Correlation between glucose concentration and immunogenicity of Amadori-albumin has also been evaluated and the study was extended with the aim to determine the presence of anti-Amadori-albumin autoantibodies in diabetic patients.

## Materials and methods

### Chemicals

Human Serum Albumin (HSA), Anti-rabbit IgG alkaline phosphatase conjugate, Protein-A agarose affinity column, p-nitrophenyl phosphate, Freund’s complete and incomplete adjuvants, were purchased from Sigma–Aldrich, USA. D-Glucose was from Sisco Research Lab (SRL), India. Flat bottom maxisorp enzyme linked immune sorbent assay (ELISA) modules were purchased from Nunc, Denmark. All other reagents/chemicals were also purchased highest analytical grade.

### Modification of HSA by glucose

Human serum albumin (HSA) (15μM) in phosphate buffer (PBS) saline, pH 7.4 was incubated in sterile condition with different concentrations of 5mM, 25mM, 50mM and 75mM of glucose for 4 days at 37°C by the protocol described elsewhere [17]. After incubation, samples were properly dialyzed with PBS, to remove excess glucose molecule and stored at -20°C till analysis. Native albumin served as control.

### Study design

Animal study was conducted in Animal house facility of J. N. Medical College, Aligarh Muslim University, Aligarh. 12 white New Zealand female rabbits, weighting 1–1.5 kg were obtained from the animal house facility of J.N. Medical College, Aligarh Muslim University, Aligarh. Rabbits were housed in the cages with wide square mesh at the bottom and placed in a well-ventilated room with 12hrs light and 12hrs dark cycle, maintained at 28±2°C temperature and 50% humidity. For one week the animals were acclimatized. All animals had a standard diet and water *ad libitum*. The experimental protocol and procedures for immunization of animals was duly standardized and approved by the Institutional Animal Ethics Committee (IAEC), J. N. Medical College, Aligarh Muslim University with approval number: 401/RO/C/2001/CPCSEA".

Rabbits were divided into three groups

Group A: 2 rabbits: Control.
Group B: 2 rabbits: Received only native albumin.
Group C: 8 rabbits in this group received Amadori-albumin (5, 25, 50, 75mM glucose modified HSA), 2 rabbits for each concentration.

### Immunization schedule and collection of sera

This study was ethically approved by institutional animal ethical committee. Rabbits were immunized with well characterized Amadori-albumin by previously described protocol [18]. Briefly, rabbits were immunized intramuscularly at multiple sites with 100μg of antigen, emulsified with an equal volume of Freund's complete adjuvant. The animals were boosted in Freund's incomplete adjuvant at weekly intervals for 6 weeks with the same amount of antigen. Pre-immune blood was collected from each animal before immunization from marginal vein. One week after the last dose of the immunogen, blood was again collected from marginal ear vein of the animals. Sera were separated and heated at 56°C for 30 min to inactivate complement proteins. Small aliquot of sera were stored at -20°C with 0.1% sodium azide as preservative. After the blood withdrawal, all animals were returned to animal care centre of JN Medical College.

### Detection of immunogenicity of anti-Amadori-albumin antibodies in experimental animal’s sera and autoantibodies in diabetic sera

Pre immune and immune sera and isolated IgG from both sera were tested for antibodies detection induced in experimental animals by direct binding ELISA, competitive ELISA and gel retardation assay. Also, sera of diabetic patients were processed to check its binding with native albumin and Amadori-albumin.

### Isolation of serum IgG on protein A-agarose column

Immunoglobulin G (IgG) was isolated by Protein A-agarose affinity column from pre-immune and immune sera [19]. 0.3 ml serum was diluted with equal volume of phosphate buffer saline (PBS, pH 7.4) and was applied on the protein A column (12mm × 45mm) equilibrated with same buffer. The wash-through fraction was recycled 2–3 times and unbound IgG was removed by extensive washing with PBS. The bound IgG was eluted with 0.58% acetic acid in 0.85% NaCl and neutralized with 1 ml of 1 M Tris-HCl, pH 8.5. Fractions (3 ml) were collected and read at 280 nm. IgG concentration was determined considering 1.4 OD_280_ = 1.0 mg/ml. The isolated IgG was then dialysed against PBS, pH 7.4, and stored at -20°C with 0.1% sodium azide. The homogeneity of isolated IgG was checked by 8% SDS-PAGE.

### Direct binding enzyme linked immunesorbent assay

Titre of the induced antibodies against native and Amadori-albumin were evaluated by direct binding ELISA. It was performed on flat bottom polysorp immuno-modules with slight modifications [20]. Briefly, plates were coated with 100μl of native or Amadori-albumin (10 μgml^-1^) for 2hrs at 37°C and overnight at 4°C. Each sample was assayed in duplicate and half of the plate, devoid of antigen coating, served as control. The plates were washed with TBS-T (20mM Tris, 150mM NaCl, pH 7.4, containing 0.05% Tween-20) and unoccupied sites were blocked with 2% fat free milk in TBS (10 mM Tris, 150 mM NaCl, pH 7.4) for 4–6 h at 37°C. After incubation, plates were washed with TBS-T, 5–6 times. The test serum serially diluted in TBS-T was added to each well (100 μl/well) and again incubated for 2hrs at 37°C and overnight at 4°C. Plate was washed 3 times and bound antibodies were assayed with anti-rabbit alkaline phosphatase conjugate using p-nitrophenyl phosphate as substrate. The plate was monitored at 410 nm by automatic ELISA plate reader. Results were expressed as a mean of A_test_- A_control_.

### Inhibition ELISA

The antigenic specificity of induced antibodies was determined by inhibition ELISA [21]. The wells were coated with 100μl of native or Amadori-albumin antigen and incubated for 2hrs at 37°C and overnight at 4°C. Varying amounts of inhibitors (0–20 μg/ml) were allowed to interact with a constant amount of antiserum or affinity purified IgG for 2hrs at 37°C and overnight at 4°C. The immune complex thus formed was added to antigen-coated plates instead of serum. The remaining steps were same as mentioned above in direct binding ELISA. Percent inhibition was calculated by using the formula:

$$\%\text{ inhibition}=\left[1-\frac{A_{inhibited}}{A_{uninhibited}}\right]\times 100$$

### Band shift assay

For the visual detection of antigen–antibody interaction, band shift assay was performed [22]. Immune complex was prepared by incubating constant amount of native or Amadori-albumin with varying amounts of affinity purified anti-native/Amadori-albumin IgG in PBS for 2h at 37°C and overnight at 4°C. The samples were then electrophoresed on 7.5% SDS–PAGE for 4hrs at 80 V and the bands were visualized by silver staining.

### Blood collection from human subjects and sera separation

Blood was collected from fifty voluntary donors with a history of type 2 diabetes attending Rajiv Gandhi Center for diabetes and endocrinology, J.N. Medical College, Aligarh Muslim University, India and from 50 healthy subjects. A detailed history on demographics and cumulative clinical and laboratory manifestations during the period of disease were recorded for every patient. The study protocol was approved by the Institutional bioethical Committee, J. N. Medical College, Aligarh Muslim University. Informed written consent was obtained from the subjects before withdrawal of the blood sample and the ethical committee approved this consent procedure. Sera, separated from the blood samples, were decomplemented by heating at 56°C for 30 min and stored in aliquots at -20°C with 0.1% sodium azide as preservative.

### Statistical analysis

Statistical analysis of the results was carried out by Student's t-test using Origin Software 6.1 (USA). Data were expressed as mean±SD. A p-value of <0.05 was considered statistically significant.

## Results

### Immunogenicity of native and Amadori-albumin

**Fig 1:**
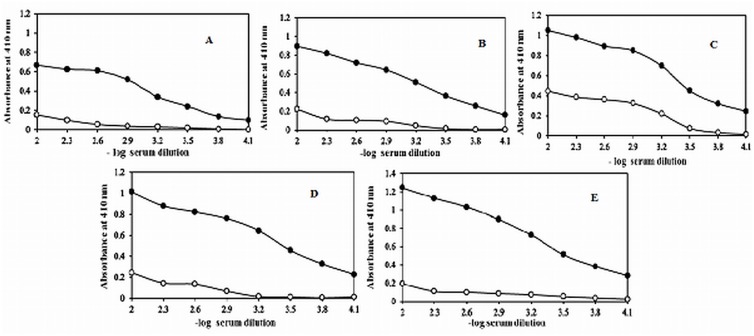
Direct binding ELISA of preimmune and immune sera. Level of induced antibodies against native and Amadori-albumin in experimental animals. Direct binding ELISA of native albumin (A) 5mM (B) 25mM (C) 50mM and (D) 75mM (E) glucose modified Amadori-albumin with preimmune (-●-) and immune sera (-ο-). The microtitre wells were coated with respective antigens (10μgml^-1^). Preimmune serum showed negligible binding with respective immunogen under identical experimental conditions.

The immunogenicity of native and Amadori-albumin was evaluated by inducing antibodies in female rabbits. Direct binding ELISA results showed induction of high titre antibodies (>1:12800) when albumin modified with 75mM glucose was used as immunogen. Antisera against native albumin and 5mM glucose modified Amadori-albumin possess almost similar, antibody response (<1:6400). Antisera against 25mM and 50mM glucose modified Amadori-albumin showed antibody titre of >1:6400 and <1:12800 respectively (Fig 1A–1D). These results demonstrate that chronic concentration of glucose (75mM) was a potent inducer of immunogenicity. This clearly indicates that the modification of HSA with chronic concentration of glucose has made the protein an even more potent immunogen. Preimmune sera showed negligible binding with respective immunogens under identical experimental conditions.

### Competitive ELISA of native and Amadori-albumin

**Fig 2:**
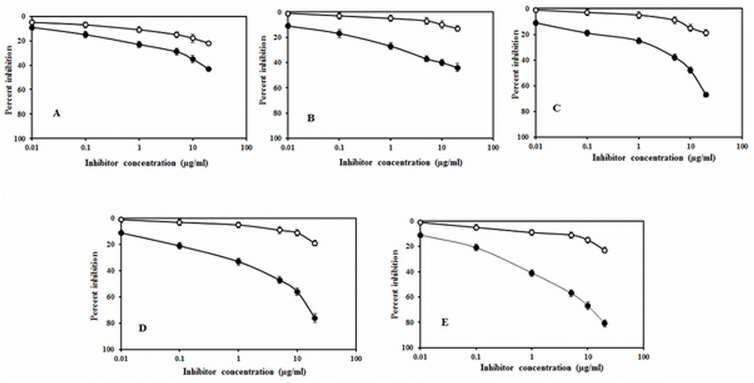
Inhibition ELISA of preimmune and immune sera. Inhibition ELISA of immune (-●-) and preimmune sera (-ο) of (A) native albumin (B) 5mM (C) 25mM (D) 50mM (E) 75mM Amadori-albumin. Microtitre plate were used as a coating antigen as well as inhibitor respectively.

Inhibition ELISA was also done to analyze the antigenic specificity of native and Amadori-albumin. A maximum of 81% inhibition in antibody binding was observed when 75mM glucose modified Amadori-albumin was used as an inhibitor. However, 5, 25, 50 mM glucose modified albumin showed a maximum inhibition of 44%, 67% and 76%, respectively at 20 μg/ml of the immunogen. A maximum of 43.2% inhibition in antibody binding was observed when native albumin was used as an inhibitor as well as coating antigen (Fig 2A–2E). 81% inhibition and lower inhibitor concentration (1.9 μg/ml) required to achieved 50% inhibition indicate that induced antibodies against Amadori-albumin (75 mM) is highly specific. Since modification of albumin by 75mM glucose induced significantly high antibody response, further immunological studies were carried out with 75mM glucose modified Amadori-albumin.

### Isolation of IgG from sera

**Fig 3:**
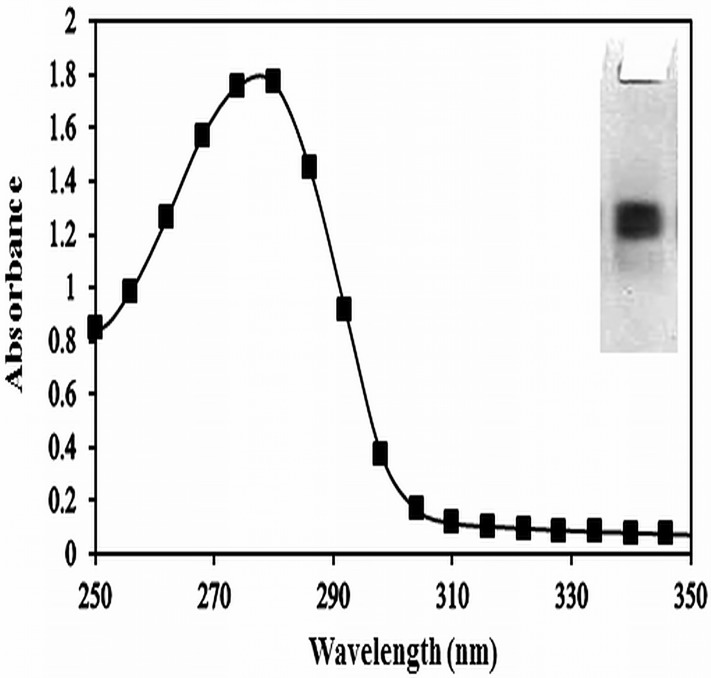
Purification of IgG. Elution profile of anti-Amadori-albumin IgG on protein-A agarose affinity column. Insert: SDS-PAGE photograph of purified IgG on 8% polyacrylamide gel.

IgG was isolated from pre-immune and immune sera by affinity chromatography on protein A agarose column. The bound IgG was eluted with 0.58% acetic acid and 0.85% sodium chloride. The peak fraction showing the ratio of 1.8 or more at A_278_/A_251_ were dialysed against PBS pH 7.4 and subjected to purity check on 8% SDS-PAGE under non reducing condition. The single band movement in SDS-PAGE confirms the homogeneity of isolated IgG (Fig 3 and Inset there in).

### Direct binding and inhibition ELISA of affinity purified IgG from anti sera

**Fig 4:**
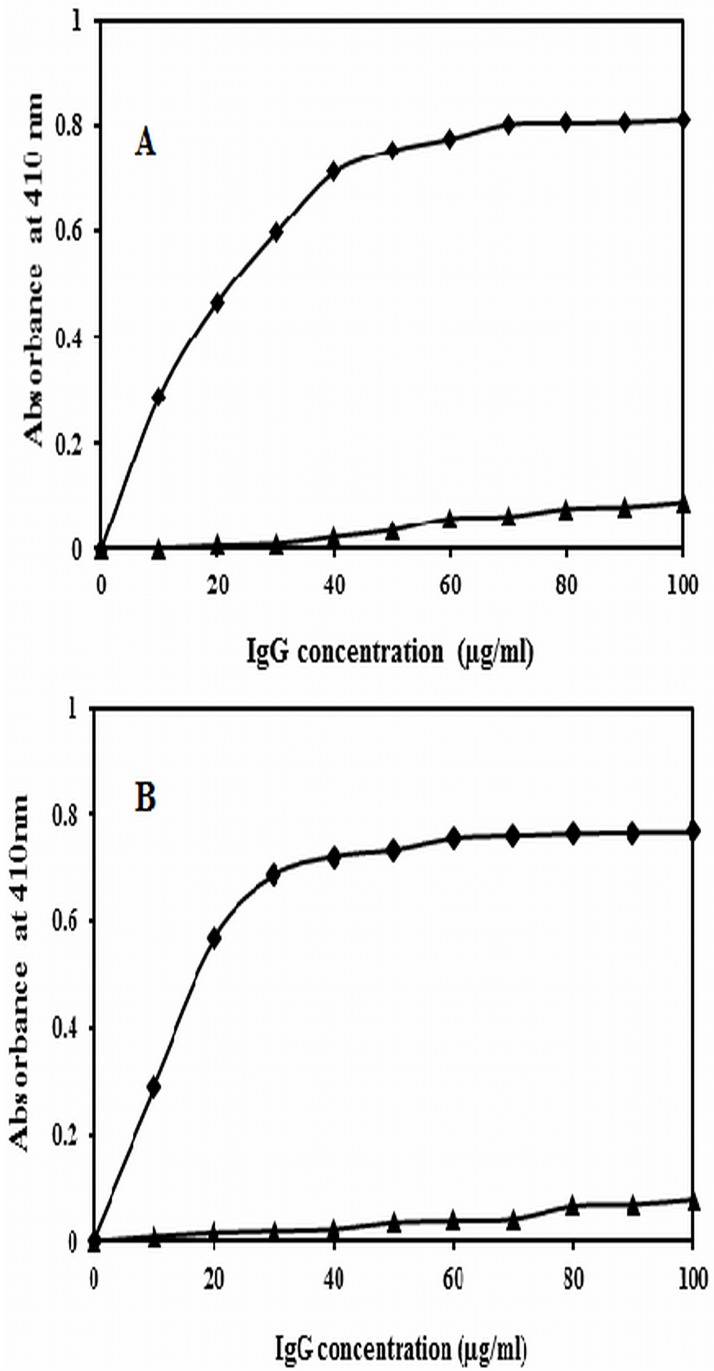
Direct binding ELISA of IgGs. Binding of immune IgG (-●-) and preimmune IgG (-ο-) with (A) native albumin (B) Amadori-albumin (75mM). Microtitre wells were coated with native albumin and Amadori-albumin (10μl/ml) respectively.

**Fig 5:**
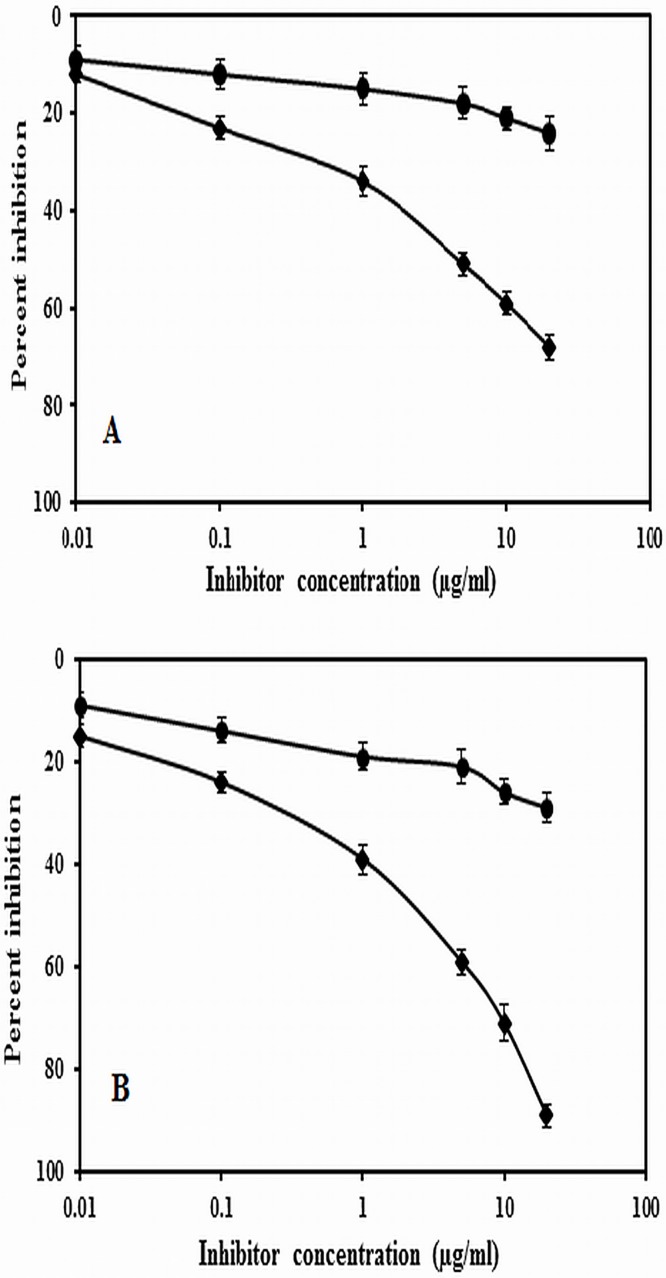
Inhibition ELISA of IgGs. Inhibition ELISA of affinity purified immune IgG (-●-) and pre-immune IgG (-ο-) (A) native albumin (B) Amadori-albumin. Native albumin and 75mM glucose modified albumin (10 μgml^-1^) was used as a coating antigen as well as inhibitor respectively.

Purified anti-native albumin IgG and anti-Amadori-albumin IgG were subjected to direct binding ELISA on microtitre plates coated with native and Amadori-albumin (75mM glucose) to evaluate the amount of antigen required for saturation. Results showed that anti-native albumin IgG and anti-Amadori-albumin IgG strongly bound to their respective immunogens. However the preimmune IgG showed negligible binding. The amount of Anti-native albumin IgG and Anti-Amadori-albumin IgG required for native albumin/ Amadori-albumin saturation was found to be at 70.2μg/ml and 45.8μg/ml, respectively (Fig 4A and 4B). The antigenic specificity of the induced anti-Amadori-albumin IgG was evaluated by inhibition ELISA. A maximum inhibition of 45.8% and 89% was observed at 20μg/ml of the native albumin and Amadori-albumin (Fig 5A and 5B). Moreover, only 3.4μg/ml of inhibitor was required to achieve 50% inhibition, thus signifying the highly specific nature of antibodies induced against 75mM glucose modified albumin.

### Analysis of epitope sharing of induced antibodies

Furthermore, cross-reactivity of anti-Amadori-albumin IgG with different inhibitors was determined to evaluate the specificity of induced antibodies. The results of cross-reactivity of anti-Amadori-albumin IgG with an array of glycated proteins/amino acid and their native analogues have been summarized in Table 1. It may be noted that Amadori proteins were preferentially bound by the experimentally induced antibodies against Amadori-albumin as compared to their native analogues. The immunogenic specificity of the experimentally induced antibodies against native albumin was also ascertained by competitive inhibition assay. Table 2 summarizes the data of the binding characteristics of anti-native-albumin IgG with native proteins/amino acids and their glycated modified counterparts.

**Table 1:** Cross-reaction of affinity purified anti-Amadori-albumin-IgG with various inhibitors as determined by inhibition ELISA.

Inhibitors	Maximum % Inhibition at 20 μg/ml	Concentration for 50% inhibition (μg/ml)	Percent relative affinity
Glycated HSA	85.3	3.3	100
Native HSA	45.8		
Native BSA	33.5		
Glycated BSA	56.4	22.8	23.5
Native human IgG	26.7		
Glycated Human IgG	45.1		
Native Histone	26.9		
Glycated Histone	43.7		
Native poly L-lysine	17.5		
Glycated poly L-lysine	49.4		
Native lysine	38.5		
Glycated lysine	74.7	12.4	33.6
Native Arginine	24.1		
Glycated Arginine	32.3		

**Table 2:** Cross-reaction of affinity purified anti-albumin IgG with various inhibitors as determined by inhibition ELISA.

Inhibitors	Maximum % Inhibition at 20 μg/ml	Concentration for 50% inhibition (μg/ml)	Percent relative affinity
Native HSA	65.3	6.3	100
Glycated HSA	55.8	19.3	
Native BSA	43.5		
Glycated BSA	32.4	22.8	23.5
Native human IgG	43.7		
Glycated Human IgG	25.4		
Native Histone	29.9		
Glycated Histone	23.4		
Native poly L-lysine	27.5		
Glycated poly L-lysine	19.9		
Native lysine	48.3		
Glycated lysine	34.1	18.4	33.6
Native Arginine	44.5		
Glycated Arginine	22.7		

### Band shift assay

**Fig 6:**
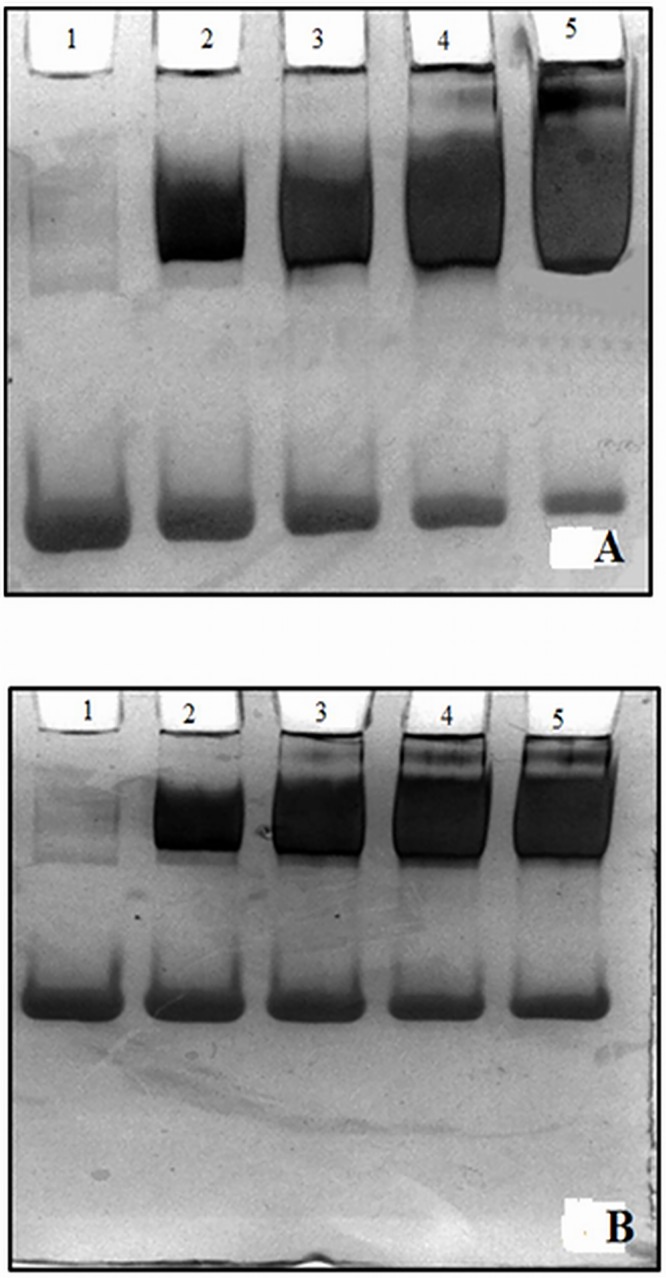
Band shift assay. Band shift assay of anti-Amadori-albumin IgG binding to (A) Amadori-albumin (75mM) and (B) native albumin. Electrophoresis was performed on 7.5% SDS-polyacrylamide gel for 4h at 80 V. (A) Amadori-albumin (25 μg/ml, lane 1) was incubated with 10, 20, 30 and 40 μg anti-Amadori-albumin-IgG respectively (lane 1–5) for 2 hr at 37°C and overnight at 4°C. (B) Native albumin was incubated with 10, 20, 30 and 40 μg anti-Amadori-albumin-IgG respectively (lane 1–5) under identical conditions.

To visualize the antibody specificity for the immunogen, gel retardation assay was performed. The antigen–antibody complexes were prepared by incubating a constant amount of native albumin and Amadori-albumin with increasing concentrations of anti-Amadori-albumin IgG and then electrophoresed at 8% SDS-PAGE. The formation of high molecular mass immune complexes between Amadori-albumin and anti-Amadori-albumin IgG with retarded mobility was observed (Fig 6A). A proportional decrease in intensity of free antigen indicates its involvement in the formation of immune complex. However, there was slight decrease in the intensity of unbound antigen when native albumin was used under identical experimental conditions (Fig 6B). The mobility shift results indicated that induced antibodies against 75mM glucose modified Amadori-albumin were highly specific but also recognize some epitopes on native albumin.

### Detection of autoantibodies against native and Amadori-albumin in diabetes patients

**Fig 7:**
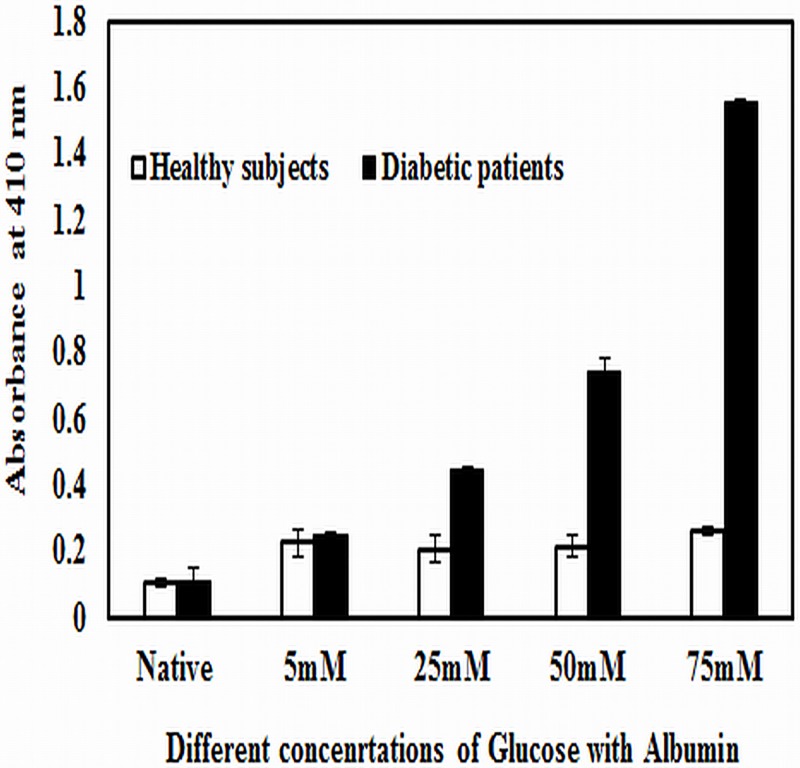
Direct binding ELISA of diabetic patients sera. Binding profile of serum autoantibodies in diabetes patients (-■-) with native albumin and Amadori-albumin modified with 5, 25, 50 and 75mM glucose. Normal human sera (-□-) served as control. The plate was coated with the respective antigens (10μg/ml). Each bar represents mean± SD of 50 healthy/ diabetic patients.

A total of 50 sera of diabetes patients were diluted 1:100 and tested for binding to native albumin and Amadori-albumin modified with different concentrations of glucose. 32% diabetes patient’s sera showed appreciably high binding with Amadori-albumin modified with 75mM glucose compared to the native albumin (Fig 7). Diabetes sera showed less or moderate binding with Amadori-albumin modified with 5, 25, 50 mM glucose. Normal sera showed negligible binding with either of the coated antigens.

## Discussion

Glucose is a ubiquitous natural metabolite present in all human tissues and reacts non-enzymatically with nucleophilic groups on proteins. Proteins modified by non-enzymatic glycation or glycoxidation processes are immunologically active and can induce significant immune response. Protein modifications in diabetes and its secondary complications may lead to Amadori as well as AGEs. Structural stability of macromolecules is the main factor to carry out all its functions, otherwise it can be involved in the development of diseases. Maryam et al reported that interaction of carbonylcyanide p-(trifluoromethoxy) phenylhydrazone (FCCP) with HSA induced structural changes on HSA [23]. During persistent hyperglycaemia, HSA a lysine rich protein can undergo fast glycation. Elaheh et al reported that glycated HSA has less binding affinity for metformin than normal HSA [24]. Our previous findings have shown that with increase in glucose concentrations, conformational changes in albumin were also increased. We have established changes in secondary and tertiary structure of albumin modified with different glucose concentrations, with the help of various techniques [17]. However, in this study we have explored the immunological properties of structurally modified albumin by different glucose concentrations. Albumin in its native form is also immunogenic [25] but due to glucose induced structural changes in albumin, it became highly immunogenic which in turn produced highly specific antibodies in experimental animal. In our experimental conditions, Amadori-albumin modified with 5mM glucose showed similar induction of antibodies as native albumin which indicate that at physiological glucose concentration HSA is not structurally perturbed. However, immunogenicity of albumin was increased as glucose concentration was increased from hyperglycaemic (25 mM and 50 mM) to chronic hyperglycaemic range (75 mM). This clearly demonstrates that an array of modifications in albumin structure depends on glucose concentration and it might favour polymerization of native epitopes of albumin into potent immunogenic neo-epitopes. Albumin modified with 75 mM glucose was found to be a potent immunogen inducing high titre antibodies in rabbits. Interpretation of our result is that glucose induced conformational alterations in structure of HSA that leads to the generation of neo-epitopes thus enhancing the immunogenicity of Amadori-albumin. Immunogenicity of modified HSA was totally dependent on glucose concentrations. Furthermore, antigenic specificity of affinity purified anti-Amadori-albumin IgG reiterated that the antibodies preferentially recognized the modified epitopes on Amadori-albumin. Notable feature of the anti-Amadori-albumin IgG was that the maximum inhibition in antibody binding was caused when Amadori-albumin (75 mM) was used as inhibitor followed by 25 mM and 50 mM and least with the 5 mM and native-albumin. Lysine residues play an important role in the enhancement of immunogenicity of albumin [26]. Many studies have reported that macromolecules undergo structural perturbations upon glycation which lead to the generation of a new form or neo-epitopes on protein that are recognized as foreign molecule by the immune system and are able to elicit antibody responses [27]. Our findings also indicate that chronic hyperglycaemic glucose concentration has caused changes in albumin structure and generated neo-epitopes on albumin that can induce immune system to produce antibodies. This suggests a role of Amadori-albumin (in chronic condition of hyperglycaemia) in the development and progression of diabetes and related complications. The induced antibodies also exhibited polyspecificity with respect to antigen binding as determined by inhibition assay. The binding of induced antibodies was tested against native and glycated forms of different proteins. It showed variable degree of recognition of the Amadori forms of other proteins. These results, therefore, indicate sharing of common epitopes between Amadori-albumin and Amadori forms of other proteins [28–29]. Moreover, anti-Amadori-albumin IgG antibodies also showed binding with native albumin. It indicates that all epitopes typical of native albumin have not been converted into neo-epitopes upon early glycation. Hence immunization with Amadori-albumin may produce polyspecific antibodies which can recognize both old and neo-epitopes. Gel retardation data further substantiated the preferential recognition of Amadori-albumin over native albumin by anti-Amadori-albumin antibodies. The formation of high molecular weight immune complex and appreciable decrease in the unbound antigen observed with Amadori-albumin, compared to native albumin, shows that the major antibodies were directed against the modified epitopes. These results indicate that native albumin binds to the anti-Amadori-albumin IgG to some extent due to the presence of native epitopes. In addition, the cross reactivity of anti-native-albumin IgG antibodies with Amadori-albumin reveals that modified HSA has got both old and neo-epitopes. Amadori-albumin was found to be immunogenic and it has been reported that type 1 diabetic patients with or without complications have higher level of Amadori-albumin [30]. Serum autoantibodies in type 2 diabetes patients showed preference of Amadori-albumin that suggests a causal role for Amadori-albumin in the pathogenesis of diabetes mellitus.

## Conclusion

Collectively, on the basis of our results, we propose that different concentrations glucose modified albumin might be playing a role in presenting unique antigenic determinants. Thus eliciting immune response that were are not ordinarily present on the native molecule, especially albumin modified with high concentration of glucose (75 mM). Changes observed in the immunological properties of Amadori-albumin might be due to conformational alterations in albumin structure upon glycation. Generation of neo-epitopes on albumin might be involved in the induction of autoantibodies in type 2 diabetes. So inhibition of Amadori-albumin production may be useful to reduce diabetes progression.
